# Recreational Exercise and Inflammatory Patterns in Hashimoto’s Thyroiditis: Observations from a Cross-Sectional Study

**DOI:** 10.3390/biom15111510

**Published:** 2025-10-25

**Authors:** Marko Vuletić, Vanna Žnidar, Ana Barić Žižić, Sanda Sladić, Dean Kaličanin, Vesela Torlak Lovrić, Maja Cvek, Ante Punda, Vesna Boraska Perica

**Affiliations:** 1 Department of Nuclear Medicine, University Hospital of Split, Spinčićeva 1, 21000 Split, Croatia; mavuletic@gmail.com (M.V.); ana.baaric@gmail.com (A.B.Ž.); sanda_gracan@yahoo.com (S.S.); veselakbsplit@yahoo.com (V.T.L.); maja.cvek.st@gmail.com (M.C.); ante.punda@gmail.com (A.P.); 2 Department of Medical Biology, University of Split School of Medicine, Šoltanska 2, 21000 Split, Croatia; vanna.znidar@mefst.hr (V.Ž.); dean.kalicanin@mefst.hr (D.K.)

**Keywords:** chemokines, CC (CCL20), chemokines, CXC (CXCL9), exercise, interleukins (IL-24), interleukin-15 receptor alpha subunit, thyroiditis

## Abstract

In this cross-sectional observational study, we investigated whether recreational exercise (RE) influences systemic inflammation in Hashimoto’s thyroiditis (HT) across different disease severity groups. We analyzed 403 participants from the Croatian Biobank of Patients with HT (CRO-HT), including 173 controls and 230 HT patients (euthyroid, levothyroxine [LT4]-treated, and hypothyroid). Serum levels of 92 inflammatory proteins were measured using the Olink^®^ Target 96 Inflammation panel, and exercise status was assessed via structured questionnaires. Linear regression revealed distinct protein associations depending on thyroid status. In controls, RE was associated with reduced MMP-10 and FGF-5, reflecting cardiovascular and muscle benefits. In euthyroid patients, RE was associated with decreased CXCL9 and TRAIL, implicating reduced type 1 inflammation and vascular risk. LT4-treated patients showed increases in IL-15RA and IL-24 with RE, suggesting improved muscle metabolism and anti-inflammatory effects. In hypothyroid patients, RE was associated with reduced CCL20 and increased HGF, while changes in TRANCE and TWEAK indicated mixed effects on bone and immune regulation. Notably, RE was associated with reduced CXCL9 and CCL20, two proteins previously linked to HT risk. Overall, RE is associated with distinct changes in inflammatory profiles across HT disease severity groups, with the most favourable responses observed in LT4-treated patients, suggesting synergy with hormone therapy.

## 1. Introduction

Hashimoto’s thyroiditis (HT) is one of the most common autoimmune disorders with marked prevalence in women. The pathogenesis of HT is characterized by lymphocytic infiltration of thyroid tissue and the production of thyroglobulin and thyroid-peroxidase antibodies (TgAb and TPOAb, respectively). Various cytokines, particularly tumor necrosis factor (TNF), interleukins (ILs), and CC chemokines, play important roles in the onset and modulation of the immune response in HT [1,2]. Chronic immune-mediated destruction of thyroid tissue leads to reduced thyroid hormone secretion that ultimately results in hypothyroidism. Although levothyroxine (LT4) replacement therapy remains the standard treatment for hypothyroidism, many patients continue to experience residual symptoms and immune dysregulation despite achieving biochemical euthyroidism [3,4]. In fact, HT is frequently associated with a broad spectrum of systemic effects, including symptom load, altered immune function, cardiovascular risk, and musculoskeletal impairment [5,6]. For this reason, many studies have focused on investigating ways to improve the overall health of patients with HT, including lifestyle modifications such as adopting regular physical activity.

Recent studies have highlighted the potential of “structured physical activity” in improving health outcomes in thyroid disease. For example, a meta-analysis of seven randomized controlled trials reported that long-term exercise interventions, when combined with standard therapy, significantly reduced serum thyroid-stimulating hormone (TSH) and increased free thyroxine (fT4) levels, suggesting that exercise may serve as an effective non-pharmacological addition to standard therapy in hypothyroidism management [7]. Moreover, regular daily physical activity was associated with improved immune parameters, such as lower levels of inflammatory markers (CRP and fibrinogen) and decreased counts of certain white blood cells in men and women from the large NANHES cohort [8]. The data on the potential anti-inflammatory effect of physical activity in HT are limited; however, the impact of regular exercise on modulating cytokine levels and other inflammatory markers was shown for various other autoimmune diseases [9,10]. In brief, physical activity was shown to increase T-regulatory cells, reduce immunoglobulin secretion, and shift the T helper type 1/T helper type 2 (Th1/Th2) balance toward lower Th1 activity. It was also found to stimulate muscle-derived IL-6, which acts as a myokine to promote anti-inflammatory effects via IL-10 induction and IL-1β inhibition [11]. A 20-year systematic review of exercise effects on autoimmune diseases supports the development of exercise intervention programs for autoimmune disease patients due to its beneficial anti-inflammatory effect [12].

Despite these findings, the molecular mechanisms through which physical activity influences inflammatory pathways in HT remain largely unknown. To address this gap, we conducted a study investigating the associations between recreational exercise (RE) and a wide range of inflammatory proteins in individuals with HT across different disease severity groups. The primary aim of this study was to examine the effect of RE on inflammatory protein levels in individuals with HT, considering both the presence and progression of the disease. The secondary aim was to determine whether RE can have beneficial effects in reducing inflammation by modulating immunopathological pathways associated with HT.

## 2. Materials and Methods

This cross-sectional observational study investigated whether RE is associated with systemic inflammation in individuals with HT by using a comprehensive Olink^®^ Target 96 Inflammation panel, a high-throughput proteomic platform enabling sensitive and multiplexed detection of immune proteins. Serum samples were obtained from the Croatian biobank of patients with Hashimoto’s thyroiditis (CRO-HT), a nationally recognized and deeply phenotyped biorepository focused on autoimmune thyroiditis in the Croatian population [13,14]. This unique resource provides an ideal foundation for integrative and translational analyses that link lifestyle factors to molecular disease signatures.

Subjects: The enrolment of study participants into the CRO-HT cohort was conducted at the Outpatient Clinic for Thyroid Disorders, Clinical Department of Nuclear Medicine at University Hospital of Split, the biggest outpatient clinic for thyroid disorders in the region, with a history of more than 60 years and averaging approximately 30,000 clinical exams per year. The recruitment process was performed by nuclear medicine specialists with an average of 17 years of experience working at the Clinical Department (range 8–32 years) [13]. Diagnosis of HT followed established clinical guidelines [15], with participants undergoing clinical and biochemical evaluation. Controls were also selected based on clinical and laboratory examinations and were required to be euthyroid with no signs of HT or other thyroid disorders. Additionally, structured questionnaires were administered to gather detailed information on personal and family history of autoimmune diseases, medical background, and various environmental and lifestyle factors, including diet, physical activity, smoking habits, and reproductive health. All participants are living in iodine-sufficient southern Croatia [16]. In summary, inclusion criteria required adult participants of white European descent with clinically confirmed HT (or absence of HT for controls), while exclusion criteria included insufficient clinical data or presence of another thyroid disorder; these criteria ensured a homogeneous population and minimized confounding. Further details on the biobank, diagnostic criteria, and phenotype collection methods are available in our published studies [14,17].

We divided HT patients into 3 disease severity groups based on the main clinical criteria: TSH levels and LT4 therapy status. Therefore, patients were divided into: (1) EUTHY group with 42 euthyroid HT patients (TSH, 0.3–3.6 mlU/L, within the normal reference range); (2) LT4 group with 88 patients receiving LT4 therapy, and (3) HYPO group with 100 hypothyroid patients (TSH > 3.6 mlU/L, above reference level). For analytical purposes, the groups were further divided into subgroups based on exercise status—those that perform exercise and those that do not (RE or Without RE).

We gathered data on RE using a self-report questionnaire consisting of three items: (1) Do you engage in sports or exercise regularly? (2) If yes, how frequently? (3) How many hours per day do you usually exercise? The scoring criteria for RE are presented in Table 1. For analytical purposes, we grouped participants into two groups: those who indicated no engagement in sports or reported only occasional sport activity were grouped in the “Without RE” group, whereas those who exercised (score 4, 8, or 16) were grouped in the “RE” group.

Blood samples were collected from all participants, and levels of TSH and thyroid hormones, including triiodothyronine (T3), thyroxine (T4), fT4, and fT3, as well as TgAb and TPOAb antibodies, were measured using LIAISON chemiluminescence immunoassays (DiaSorin Saluggia, Saluggia, Italy).

The Olink Target 96 Inflammation panel was used to measure 92 inflammatory proteins using the proximity extension assays (PEA) from the stored serum (at −80 °C). The protein levels were expressed in Normalized Protein Expression (NPX) values, reflecting the relative abundance of each protein. Samples were successfully measured, with 95% passing quality control. Details on PEA technology can be found in a published study [18].

Statistical analyses. To assess differences in median RE values between the subgroups within each severity group, a Kruskal–Wallis test was performed, followed by pairwise Wilcoxon rank sum tests. Linear regression models were used to examine the association between NPX levels and exercise status (RE and Without RE), adjusting for variables including age, gender, and body mass index (BMI), within each group (Control, EUTHY, LT4, HYPO). For each assay, the models were fitted separately, and *p*-values were calculated for each predictor, with *p*-values adjusted using the Benjamini–Hochberg method to control for multiple comparisons.

## 3. Results

**Figure 1:**
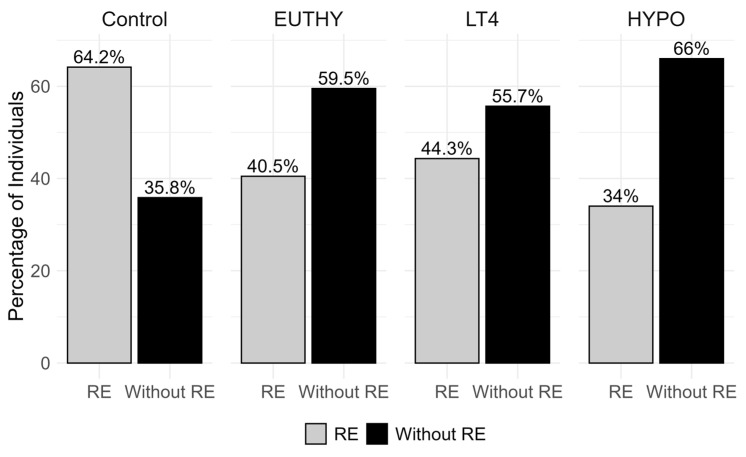
Distribution of individuals engaged in recreational exercise (RE; grey) and those not engaged (Without RE; black) across the four study groups: Control, euthyroid HT (EUTHY), LT4-treated HT (LT4), and hypothyroid HT (HYPO). Values represent the percentage of individuals within each group.

A total of 403 individuals were included in the study, comprising 230 participants with HT (93% female) and 173 control participants (94.2% female). Clinical characteristics of the Control group and the three groups of patients are shown in Table 2. The distribution of individuals who perform exercise and those who do not (RE or Without RE), across all groups, is shown in Figure 1. The proportion of individuals engaged in RE is highest in the Control group and decreases toward the HYPO group, while the opposite trend is observed for non-exercisers, with the lowest proportion in Controls and the highest in HYPO (Figure 1). In line, the Kruskal–Wallis test shows a statistically significant difference in the median RE values among all four groups (χ^2^ = 12.025, df = 3, *p* = 0.0073), while pairwise comparisons identified a significant difference between the Control and HYPO (*p* = 0.0043), while no significant differences are found between the other pairwise comparisons.

In Table 3, we present significant proteins that are differentially expressed between individuals who engage in RE and those who do not, across 4 groups. In Controls, we observed a modest reduction in circulating levels of two proteins in healthy individuals who are physically active compared to non-active controls: matrix metalloproteinase-10 (MMP-10) with a 1.04-fold decrease in mean NPX and fibroblast growth factor 5 (FGF-5) with a 1.36-fold decrease in mean NPX. In physically active EUTHY patients, we observed a modest reduction in three proteins: already mentioned MMP-10 with a 1.055-fold decrease in mean NPX, TNF-related apoptosis-inducing ligand (TRAIL) with a 1.028-fold decrease in mean NPX, and C-X-C motif chemokine ligand 9 (CXCL9) with a 1.07-fold reduction in mean NPX. In physically active LT4 patients, we observed a marked increase in circulating levels of two proteins: interleukin 15 receptor subunit alpha (IL-15RA) with a 1.54-fold increase in mean NPX and interleukin 24 (IL-24) with a bold 5.3-fold increase in mean NPX. In physically active HYPO patients, we observed an increase in circulating levels of three proteins: TNF-related weak inducer of apoptosis (TWEAK) with a 1.028-fold increase in mean NPX, tumor necrosis factor ligand superfamily member 11 (TNFSF11 or TRANCE) with a 1.063-fold increase in mean NPX values, and hepatocyte growth factor (HGF) with a 1.016-fold increase in mean NPX values. We also observed a decrease in C-C motif chemokine ligand 20 (CCL20) with a 1.075-fold decrease in mean NPX values.

**Figure 2:**
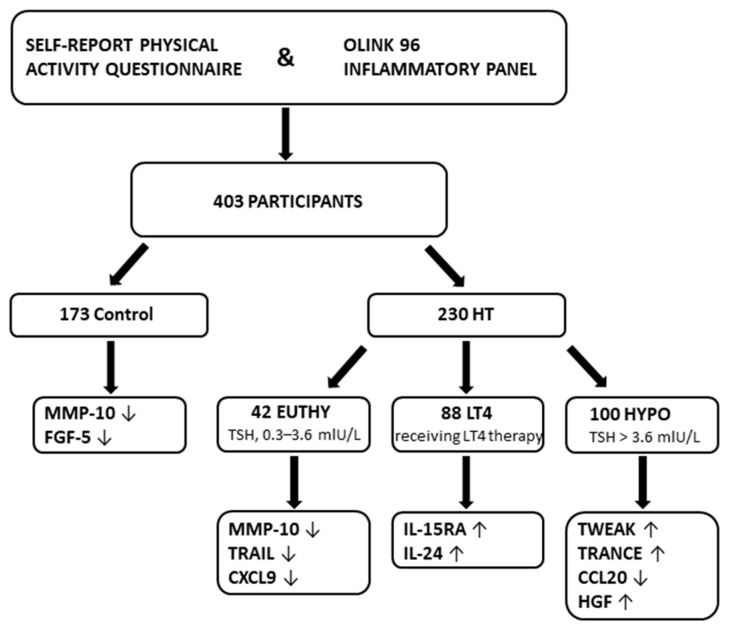
A schematic figure illustrating the study design and the main findings. Upward (↑) and downward (↓) arrows indicate the direction of the association (regression estimate) between recreational exercise (RE) and protein levels. Specifically, ↑ denotes higher protein levels in individuals engaging in RE compared to those without RE, while ↓ denotes lower protein levels.

Only proteins with statistically significant adjusted *p*-values were included in the main results, while the results for all 92 proteins across 4 groups are provided in Supplementary Materials Table S1. To enhance clarity and improve visualization of the study results, we have now introduced a schematic figure illustrating the study design and the main findings (Figure 2). All procedures and reporting in this study were conducted in accordance with the STROBE (Strengthening the Reporting of Observational Studies in Epidemiology) guidelines for cross-sectional observational studies (Supplementary Materials Table S2).

## 4. Discussion

The hypothesis of our study is that RE may be associated with systemic inflammation and immune function in individuals with HT, potentially contributing to overall health benefits, with effects varying according to disease severity and treatment status. Through the analysis of 92 inflammation-related proteins in individuals from the CRO-HT biobank, we identified distinct proteins associated with RE in both healthy subjects and HT patients, stratified according to disease severity. Many identified proteins overlap with molecular pathways involved with cardiovascular regulation, musculoskeletal health, and thyroid autoimmunity.

Regulatory T cells (Tregs) are known to have a role in maintaining homeostasis and maintaining tolerance of autoimmune conditions [19]. The working muscles release potent myokines like IL-6, PGC1α (PPARγ coactivator-1 α), myostatin, transforming growth factor β (TGF-β) superfamily, IL-15, brain-derived neurotrophic factor (BDNF), and others that are involved in immunological anti-inflammatory processes [19,20]. Exercise leads to the induction of a functional and stable Treg phenotype contributing to overall immune tolerance [19]. Introduction of physical activity has shown beneficial effects through the maintenance of low inflammatory response in various heterogeneous diseases, including Alzheimer’s disease [21], and also contributing to better immunotherapy response in cancer treatment through modulation of the tumor immune microenvironment [22].

Effects of regular physical activity in autoimmune diseases have also been studied over the years and have shown that habitual physical activity modulates cytokine levels and other inflammatory markers associated with autoimmunity [11,23]. A recent study by Patterson et al. [24] shows exacerbated pathologic inflammatory signaling in physically inactive individuals with systemic lupus. Another study suggests physical activity may contribute to slowing down of beta-cell destruction in type 1 diabetes, helping preserve pancreatic function [25].

In essence, regular physical activity has anti-inflammatory and immune-modulating effects at the cellular level, promoting homeostasis, reducing chronic inflammation, enhancing immune balance, and preventing the cellular dysfunction that underlies aging and lifestyle-related diseases [26]. However, despite many studies regarding physical activity and its effects on overall health, there have not been many studies, particularly involving inflammatory markers in autoimmune thyroid disease. There is only a study by Klasson et al. [8] regarding physical activity and immune system markers and thyroid: even so, immune function was assessed from circulating C-reactive protein (CRP), immunoglobulin E (IgE), fibrinogen levels, and blood cell counts. To our knowledge, our study is the first comprehensive study analyzing physical activity with inflammatory protein markers in HT.

Comparison with HT-Risk Proteins: To gain deeper insight into the potential immunomodulatory effects of physical activity, we compared our findings with those from our previous study conducted on the same cohort, which identified proteins associated with the risk of developing HT. Notably, two proteins—CXCL9 and CCL20—previously linked to increased risk for HT in our CRO-HT biobank (manuscript under review, shown in Supplementary Materials Table S3) are also significantly associated with RE status in our current analysis. Interestingly, both exhibited opposite effects: they showed upregulation with the advancement of HT but also downregulation associated with physical activity. Also, one of the proteins with the greatest fold increase, IL-24, is known for its tolerogenic properties in autoimmunity [27]. Therefore, our results highlight the potential of physical activity to counteract autoimmune processes relevant to HT.

In the text below, we discuss all our findings in the light of the main functions of significant proteins, with a focus on their immunological and other physiological functions potentially influenced by physical activity.

Controls: MMP-10 is a zinc-dependent endopeptidase involved in extracellular matrix (ECM) degradation and in physiological processes like development, reproduction, and tissue remodeling [28]. While MMP-10 has not been directly linked to physical activity, MMP-2 and MMP-9 were observed to decrease with aerobic exercise and weight loss, indicating reduced inflammation and ECM activity [29]. On the contrary, elevated MMP-10 is associated with cardiovascular disease (CVD), promoting inflammation, plaque formation, and heart failure progression [30,31,32]. To summarise, our data show that exercise is associated with MMP-10 reduction that may potentially contribute to cardiovascular protection, reduced inflammation, and reduced ECM. The same effect is also seen in our euthyroid HT patients undergoing RE.

FGF-5 is a glycosaminoglycan-binding protein that plays roles in embryonic development, cell proliferation, morphogenesis, tissue repair, and tumor progression. It is particularly relevant to skeletal muscle, where it promotes fibroblast proliferation and inhibits muscle development [33], modulates metabolism during myogenesis [34], and suppresses muscle fiber growth, as shown in FGF5 knockout models [35]. Its reduction with exercise may support muscle hypertrophy and improved skeletal-muscular balance by reducing fibroblast proliferation and shifting metabolism towards greater energy efficiency during physical activity. Our findings are the first to indicate potential modulation of FGF-5 with physical activity. On the other side, a more commonly studied protein in the context of exercise, FGF-21 [36,37,38], did not show a significant change in our cohort (Supplementary Materials Table S1).

EUTHY: TRAIL is a cytokine that induces apoptosis in tumor cells, and plays a role in vascular biology, exhibiting anti-inflammatory and anti-atherosclerotic effects [39]. Clinically, lower TRAIL levels are linked to higher CVD risk and mortality [40,41]. While little is known about TRAIL’s response to physical activity, one study in obese individuals found no significant change following long-term exercise [42]. Our finding of decreased TRAIL with RE is unexpected, given exercise’s cardiovascular benefits, and may reflect unique immune or metabolic dynamics in euthyroid HT, a population already at increased cardiovascular risk [6,43]. Further research is needed to clarify the mechanisms underlying this inverse relationship.

CXCL9 is a pro-inflammatory cytokine induced by interferon-gamma and is central to type 1 (T1) inflammatory responses [44]. Elevated CXCL9 levels have been linked to reduced musculoskeletal and physical function, lower activity levels, and greater frailty in older adults [45,46,47], while exercise has been shown to lower CXCL9 and improve metabolic and immune parameters in obesity [48]. As already pointed out, we previously observed a marked increase in CXCL9 levels in patients who progress from an euthyroid to a hypothyroid state. These findings suggest that physical activity may help regulate CXCL9 signaling, potentially improving musculoskeletal function and reducing chronic inflammation, and delaying hypothyroidism in HT.

LT4 group: IL-15RA is a pro-inflammatory cytokine receptor that binds IL-15 with high affinity, promoting activation of CD8+ T cells and NK cells [49,50]. It also regulates IL-15 production, secretion, and stability [51]. Beyond its role in immunology, IL-15 is involved in muscle metabolism, contributing to fat loss, improved insulin sensitivity, and reduced hepatic steatosis [52,53]. Consistent with our results, a meta-analysis showed that acute, but not chronic, exercise increases IL-15 levels shortly after activity [54]. Our results suggest that increased IL-15RA may contribute to enhanced metabolic outcomes in physically active LT4-treated patients.

IL-24 is a member of the IL-10 cytokine family with both pro- and anti-inflammatory roles and roles in tumor suppression [55]. IL-24 is also implicated in autoimmunity, where it helps regulate pathogenic T helper type 17 (Th17) responses that may aid in resolving tissue inflammation [27]. To date, IL-24 has not been investigated in the context of physical activity, rendering this a novel finding that highlights its potential role as a mediator of RE–induced immunoregulation.

HYPO group: TWEAK is a cytokine from the TNF ligand superfamily that exists in soluble and membrane-bound forms and is involved in apoptosis, inflammation (e.g., IL-8 induction), and angiogenesis via endothelial cell proliferation and migration [56,57,58]. Regular physical activity has been shown to increase circulating soluble TWEAK (sTWEAK), potentially helping balance pro- and anti-inflammatory responses [59]. However, TWEAK was also found to impair skeletal muscle by reducing mitochondrial content and angiogenesis [60,61]. Thus, in skeletal muscle tissue, TWEAK suppresses angiogenesis and promotes muscle wasting and impaired regeneration. This contrasts with its pro-angiogenic role seen in other tissues or contexts. These findings, along with our data, highlight TWEAK’s complex, context-dependent roles in inflammation and metabolism, warranting further study in hypothyroid populations undergoing exercise.

TRANCE regulates immune responses and bone resorption by promoting osteoclast differentiation via receptor activator of nuclear factor kappa-B (RANK), counterbalanced by osteoprotegerin (OPG), which inhibits this interaction [62,63]. Disruption of the TRANCE–OPG axis—via elevated TRANCE or reduced OPG—contributes to bone loss, as seen in postmenopausal osteoporosis [64,65]. Although we observe a modest TRANCE increase with RE, we also observe a non-significant decrease in OPG and OPG/TRANCE ratio, suggesting a non-pathological TRANCE increase. With respect to exercise, a decrease in TRANCE is common [66], although it may vary by sex and age [67]. Thus, the modest TRANCE increase observed in our physically active hypothyroid patients may not be pathologic, but rather restorative, especially if balanced by unchanged or mildly decreased OPG.

CCL20 is a chemokine that plays a central role in immune regulation by recruiting immature dendritic cells, memory T cells, and B cells via CCR6 [68]. As already mentioned, the CCL20 increase is associated with HT (manuscript under review). A key aspect of HT immunopathology involves Th17 cells [69], and emerging evidence suggests that CCL20 signaling can promote the conversion of regulatory T cells (Tregs) into pathogenic Th17 cells [70]. Thus, the observed reduction in CCL20 with RE may reflect exercise-induced mitigation of Th17-driven inflammation, particularly evident in hypothyroid HT patients. This aligns with prior studies reporting decreased CCL20 levels after physical activity, supporting its role in lowering systemic inflammation [71,72].

HGF is a pleiotropic growth factor that promotes epithelial proliferation, angiogenesis, and tissue repair, while also exerting anti-inflammatory and anti-apoptotic effects, particularly in the heart and brain [73]. It supports bone regeneration through enhanced osteoblast activity and neovascularization, though it may also influence bone resorption under specific conditions [74,75]. Exercise is known to transiently elevate HGF levels [76,77], which aligns with our study and suggests that this response may aid cardiovascular, muscular, vascular, and bone health in hypothyroid individuals.

Taken together, this study offers compelling evidence that RE modulates inflammatory proteins involved in HT, with potential benefits extending to autoimmune regulation, cardiovascular health, bone metabolism, and muscle maintenance. Below, we summarise our findings and discuss them by integrating the roles of individual proteins with the effects of physical activity, highlighting their interconnected impact on multiple physiological processes.

Immunomodulation and autoimmunity: RE was associated with the downregulation of proinflammatory chemokines CXCL9 and CCL20, both linked to HT risk in our previous study. The upregulation of IL-24, a cytokine with anti-inflammatory and immunoregulatory roles in Th17 cells, supports a broader anti-autoimmune shift potentially induced by RE.

Cardiometabolic regulation: Exercise-induced changes in MMP-10 and HGF point to improvements in cardiovascular and metabolic profiles. The downregulation of MMP-10, a protease involved in vascular inflammation, and upregulation of HGF, a tissue-reparative and angiogenic factor, suggest a cardioprotective shift. Although TRAIL showed a paradoxical decrease, its interpretation may require further investigation within the unique immunometabolic context of euthyroid HT.

Skeletal muscle and metabolic adaptation: The observed decrease in FGF-5, a known inhibitor of muscle growth and regulator of fibroblast activity, alongside an increase in IL-15RA, which promotes muscle metabolism and insulin sensitivity, suggests that RE supports muscle hypertrophy and metabolic efficiency—particularly in LT4-treated patients. Reduced CXCL9 also associates with musculoskeletal benefits.

Osteoimmunological Crosstalk: Proteins such as TRANCE, CCL20, and HGF—each with dual roles in inflammation and bone remodeling—responded to RE in HT patients, highlighting the intersection between immune and skeletal systems. These findings reinforce the concept of osteoimmunology, particularly relevant in hypothyroid patients, where both bone metabolism and immune balance are dysregulated.

Severity of HT: Finally, we also observe differences in inflammatory profiles associated with RE depending on the disease severity group. For example, in healthy controls, RE led to favorable but expected changes, such as reductions in MMP-10 and FGF-5, reflecting improved cardiovascular and muscular profiles. However, since these participants are euthyroid and disease-free, the clinical significance of these changes is relatively limited compared to the HT subgroups. The LT4 group demonstrates the clearest, most favorable, and least ambiguous molecular responses to RE—spanning metabolic, muscular, and immunoregulatory domains. These responses suggest that exercise acts synergistically with hormone replacement, potentially restoring a more balanced physiological state than either treatment alone. In contrast, euthyroid HT patients exhibited a potentially adverse drop in TRAIL, which may reflect a vulnerable state—HT pathology without hormonal compensation (as in LT4). Untreated hypothyroid patients showed mixed responses—some beneficial (increase in HGF and decrease in CCL20), others ambiguous (increase in TWEAK and in TRANCE), raising concerns for bone health and inflammation. These findings highlight group-specific effects of exercise, with LT4-treated individuals emerging as the most responsive and physiologically balanced subgroup.

Advantages and limitations: The use of the same well-defined cohort across both the current RE-focused analysis and a previous study on HT risk enables a unique within-subject comparison, revealing biologically meaningful and opposing trends in key proteins such as CXCL9 and CCL20. However, certain limitations should be acknowledged. The observational nature of the study precludes causal inference, and the absence of longitudinal or functional clinical outcomes limits interpretation regarding long-term or symptomatic benefits. Additionally, although the analyses were adjusted for age, sex, and BMI, other potential confounding factors, such as psychiatric history, medication use, and dietary habits, may have influenced systemic inflammation and could not be fully controlled for. Therefore, future analyses should take all of these factors into consideration to better delineate the independent associations of recreational exercise and inflammatory proteins. Finally, our findings require validation in independent cohorts.

## 5. Conclusions

In our study, we identified several proteins associated with cardiovascular and metabolic regulation (MMP-10, HGF, TRAIL, IL-15RA) as well as skeletal and immune systems (TRANCE, CCL20, HGF), demonstrating that the physiological phenotype of RE is reflected at the molecular level. The opposing regulation of HT-risk proteins CXCL9 and CCL20 by RE is particularly noteworthy, supporting the hypothesis that physical activity may help mitigate autoimmune progression. Additionally, the most favorable molecular responses were observed in LT4-treated patients, suggesting potential synergistic effects between hormone replacement therapy and physical activity.

These findings have both practical and scientific implications. Clinically, they support the potential benefits of incorporating RE into the management of HT to improve immune regulation, cardiovascular and skeletal health, and metabolic function. From a research perspective, the specific proteins identified (such as IL-24, IL-15RA, CXCL9, and CCL20) may serve as biomarkers to monitor exercise-induced immunomodulation and provide insight into mechanisms linking physical activity with autoimmune and metabolic pathways.

Future directions should focus on confirming these associations in larger and longitudinal studies, examining how different intensities or durations of RE influence inflammatory and immune biomarkers, and validating the role of key proteins identified as potential mediators of exercise-induced benefits in HT.

Collectively, these efforts will help position this study within a broader research context and guide the development of targeted exercise interventions to optimize health outcomes in patients with HT.

## Figures and Tables

**Table 1:** Calculation of the score for recreational exercise (RE).

	Less Than an Hour	Between 1–2 h	Over 2 h
Daily	16	16	16
2–3 times a week	8	16	16
Once a week	4	8	8
Occasionally	0	0	0

**Table 2:** Clinical characteristics of controls and HT patients across three disease severity groups.

Phenotype	Control	EUTHY	LT4	HYPO
	*n* = 173	*n* = 42	*n* = 88	*n* = 100
	Mean (SD)	Mean (SD)	Mean (SD)	Mean (SD)
Age, years	39.24 (11.88)	34.39 (12.67)	40.48 (13.87)	39.05 (13.22)
BMI, kg/m^2^	23.58 (3.92)	23.80 (3.80)	24.21 (4.25)	24.19 (4.22)
T3, nmol/L	1.55 (0.22)	1.62 (0.42)	1.64 (0.32)	1.51 (0.39)
T4, nmol/L	102.92 (20.30)	98.52 (22.92)	114.47 (26.78)	94.18 (27.05)
fT4, pmol/L	12.89 (1.62)	12.64 (1.53)	13.09 (2.40)	10.50 (2.50)
TSH, mIU/L	1.61 (0.67)	2.11 (0.96)	4.89 (8.78)	15.89 (24.93)
TgAb, IU/mL	17.36 (17.46)	730.52 (1177.64)	575.48 (1126.23)	618.80 (1048.81)
TPOAb, IU/mL	5.73 (5.88)	522.37 (483.10)	546.01 (575.37)	620.51 (692.42)
RE, hours/month	5.80 (5.68)	4.57 (6.34)	5.45 (6.79)	4.00 (6.15)

BMI—body mass index; T3—triiodothyronine; T4—thyroxine; fT4—free thyroxine; TSH—thyroid-stimulating hormone; TgAb—thyroglobulin antibodies; TPOAb—thyroid peroxidase antibodies; RE—recreational exercise; *n*—number of participants; SD—standard deviation; Control—healthy subjects without Hashimoto’s thyroiditis; EUTHY—Hashimoto’s thyroiditis patients with euthyroid function; LT4—Hashimoto’s thyroiditis patients receiving levothyroxine therapy; HYPO—Hashimoto’s thyroiditis patients with untreated hypothyroidism.

**Table 3:** Significant Associations Between Inflammatory Proteins and Recreational Exercise Across Study Groups.

Assay	Estimate *	*p*-value	adjusted *p*-value
Control (RE = 111, Without RE = 62)			
MMP-10	−0.4133	0.0026	0.0065
FGF-5	−0.0957	0.0058	0.0292
EUTHY (RE = 17, Without RE = 25)			
MMP-10	−0.5335	0.0070	0.0175
TRAIL	−0.2298	0.0150	0.0375
CXCL9	−0.4858	0.0165	0.0412
LT4 (RE = 39, Without RE = 49)			
IL-15RA	0.1958	0.0042	0.0126
IL-24	0.4020	0.0098	0.0163
HYPO (RE = 34, Without RE = 66)			
TWEAK	0.2526	0.0019	0.0047
TRANCE	0.3096	0.0272	0.0340
CCL20	−0.4781	0.0218	0.0364
HGF	0.1936	0.0275	0.0459

* The estimate for exercise status represents the difference in Normalized Protein Expression (NPX) levels between individuals who engage in recreational exercise (RE) and those who do not (Without RE). Adjusted *p*-values < 0.05 were considered statistically significant. Control—healthy subjects without Hashimoto’s thyroiditis; EUTHY—Hashimoto’s thyroiditis patients with euthyroid function; LT4—Hashimoto’s thyroiditis patients receiving levothyroxine therapy; HYPO—Hashimoto’s thyroiditis patients with untreated hypothyroidism.

## Data Availability

The data supporting the conclusions of this article will be made available by the corresponding author upon request.

## Supplementary Materials

The following supporting information can be downloaded at: https://www.mdpi.com/article/10.3390/biom15111510/s1, Table S1: The results of the linear regression model between NPX levels of 92 inflammatory proteins and RE status across 4 groups; Table S2: STROBE Checklist for cross-sectional studies; Table S3: The results of ANOVA analysis between NPX values and HT-disease status.
