# Supplementary material for: Recreational Exercise and Inflammatory Patterns in Hashimoto’s Thyroiditis: Observations from a Cross-Sectional Study
Source: Biomolecules. 2025 Oct 25;15(11):1510. doi: 10.3390/biom15111510 (PMC12650045; doi:10.3390/biom15111510)
Supplement: Supplementary file 1 [file biomolecules-15-01510-s001.zip › Supplementary Table S2.pdf]

STROBE Statement—Checklist of items that should be included in reports of ***cross-sectional studies***

	Item No	Recommendation	Page/Section in Manuscript
**Title and abstract**	1	(*a*) Indicate the study's design with a commonly used term in the title or the abstract	Title, p. 1: "A Cross-Sectional Study" clearly indicates study design
		(*b*) Provide in the abstract an informative and balanced summary of what was done and what was found	Abstract, p. 1: structured and includes objectives, methods, main findings, and conclusions
**Introduction**			
Background/rationale	2	Explain the scientific background and rationale for the investigation being reported	Introduction (p. 1–2): provides detailed background on Hashimoto's thyroiditis, its immunopathogenesis, and rationale for investigating the role of physical activity
Objectives	3	State specific objectives, including any prespecified hypotheses	End of Introduction, p. 2: clearly states primary and secondary study aims regarding the association between recreational exercise and inflammatory protein profiles in HT
**Methods**			
Study design	4	Present key elements of study design early in the paper	Materials and Methods, first paragraph (p. 2): cross-sectional observational study clearly stated
Setting	5	Describe the setting, locations, and relevant dates, including periods of recruitment, exposure, follow-up, and data collection	Materials and Methods, "Subjects" section (p. 2–3): University Hospital of Split; recruitment and biobank description provided
Participants	6	(*a*) Give the eligibility criteria, and the sources and methods of selection of participants	Materials and Methods, "Subjects" (p. 2–3): inclusion and exclusion criteria, clinical evaluation, selection of controls
Variables	7	Clearly define all outcomes, exposures, predictors, potential confounders, and effect modifiers. Give diagnostic criteria, if applicable	Materials and Methods, "Subjects" + "Statistical analyses" (p. 3): thyroid hormones, antibody levels, RE score, adjustments for age, sex, BMI
Data sources/ measurement	8*	For each variable of interest, give sources of data and details of methods of assessment (measurement). Describe comparability of assessment methods if there is more than one group	Materials and Methods (p. 3): LIAISON immunoassay for thyroid hormones; Olink® Target 96 Inflammation panel for proteins; same procedures for all participants
Bias	9	Describe any efforts to address potential sources of bias	Materials and Methods, "Statistical analyses" (p. 3): adjustment for age, sex, BMI; quality control (95% pass rate) for Olink assays
Study size	10	Explain how the study size was arrived at	Materials and Methods, "Subjects" (p. 2–3): based on available CRO-HT

			cohort (n=403); all eligible samples included
Quantitative variables	11	Explain how quantitative variables were handled in the analyses. If applicable, describe which groupings were chosen and why	Materials and Methods, "Statistical analyses" (p. 3): RE dichotomized (RE vs. Without RE) based on questionnaire scoring
Statistical methods	12	(*a*) Describe all statistical methods, including those used to control for confounding	Materials and Methods, "Statistical analyses" (p. 3): linear regression adjusted for age, sex, BMI
		(*b*) Describe any methods used to examine subgroups and interactions	Materials and Methods, "Statistical analyses" (p. 3): analyses performed separately within four disease severity groups (Control, EUTHY, LT4, HYPO)
		(*c*) Explain how missing data were addressed	Not applicable (participants with incomplete data were not included in the dataset)
		(*d*) If applicable, describe analytical methods taking account of sampling strategy	Not applicable (entire available cohort used, not a sampled subset)
		(*e*) Describe any sensitivity analyses	Not applicable (no sensitivity analysis performed)
**Results**			
Participants	13*	(a) Report numbers of individuals at each stage of study—eg numbers potentially eligible, examined for eligibility, confirmed eligible, included in the study, completing follow-up, and analysed	Results, p. 3: "A total of 403 individuals were included… 230 HT patients and 173 controls."
		(b) Give reasons for non-participation at each stage	Not applicable (final dataset included only participants with complete data)
		(c) Consider use of a flow diagram	Included as Figure 2
Descriptive data	14*	(a) Give characteristics of study participants (eg demographic, clinical, social) and information on exposures and potential confounders	Results (p. 3–4): Table 2 provides age, BMI, thyroid function parameters, and antibodies
		(b) Indicate number of participants with missing data for each variable of interest	Not applicable (only complete cases were analyzed)
Outcome data	15*	Report numbers of outcome events or summary measures	Results (p. 4–5): Table 3 summarizes significant associations (proteins, estimates, and p-values)
Main results	16	(*a*) Give unadjusted estimates and, if applicable, confounder-adjusted estimates and their precision (eg, 95% confidence interval). Make clear which confounders were adjusted for and why they were included	Methods, p. 3 and Results, p. 4: linear regression adjusted for age, sex, and BMI

		(*b*) Report category boundaries when continuous variables were categorized	Methods, p. 3: disease severity defined by TSH thresholds (EUTHY: 0.3–3.6 mIU/L; HYPO: > 3.6 mIU/L)
		(*c*) If relevant, consider translating estimates of relative risk into absolute risk for a meaningful time period	Not applicable (no risk estimates presented)
Other analyses	17	Report other analyses done—eg analyses of subgroups and interactions, and sensitivity analyses	Results (p. 4–5): subgroup analyses by disease severity (Control, EUTHY, LT4, HYPO)
**Discussion**			
Key results	18	Summarise key results with reference to study objectives	Discussion (p. 5–9): results are explicitly summarized with respect to the study aims, highlighting protein changes across HT severity groups and their biological significance
Limitations	19	Discuss limitations of the study, taking into account sources of potential bias or imprecision. Discuss both direction and magnitude of any potential bias	Discussion, p. 9 — includes detailed discussion of design limitations (observational nature, potential confounding factors, absence of longitudinal data)
Interpretation	20	Give a cautious overall interpretation of results considering objectives, limitations, multiplicity of analyses, results from similar studies, and other relevant evidence	Discussion (p. 8–9): interpretation integrates findings with existing evidence, notes biological plausibility, and avoids overgeneralization
Generalisability	21	Discuss the generalisability (external validity) of the study results	Discussion & Conclusions, p.9: explicitly notes that results are from a well-characterized Croatian cohort and require validation in independent populations
**Other information**			
Funding	22	Give the source of funding and the role of the funders for the present study and, if applicable, for the original study on which the present article is based	Funding, p. 9: *The formation of the CRO-HT biobank and measurements of Olink proteins were funded by the Croatian Science Foundation under projects UIP-11-2013-4950 and IP-2022-10-4071*

*Give information separately for exposed and unexposed groups.

Note: An Explanation and Elaboration article discusses each checklist item and gives methodological background and published examples of transparent reporting. The STROBE checklist is best used in conjunction with this article (freely available on the Web sites of PLoS Medicine at http://www.plosmedicine.org/, Annals of Internal Medicine at http://www.annals.org/, and Epidemiology at http://www.epidem.com/). Information on the STROBE Initiative is available at www.strobe-statement.org.
